# Engineering a *Streptomyces coelicolor* biosynthesis pathway into *Escherichia coli* for high yield triglyceride production

**DOI:** 10.1186/s13068-014-0172-0

**Published:** 2014-12-24

**Authors:** Santiago Comba, Martín Sabatini, Simón Menendez-Bravo, Ana Arabolaza, Hugo Gramajo

**Affiliations:** Microbiology Division, IBR (Instituto de Biología Molecular y Celular de Rosario), Consejo Nacional de Investigaciones Científicas y Técnicas, Facultad de Ciencias Bioquímicas y Farmacéuticas, Universidad Nacional de Rosario, Ocampo y Esmeralda (2000), Rosario, Argentina

**Keywords:** TAG biosynthesis, Phosphatidate phosphatase, *Escherichia coli*, Oil production, Triacylglycerol

## Abstract

**Background:**

Microbial lipid production represents a potential alternative feedstock for the biofuel and oleochemical industries. Since *Escherichia coli* exhibits many genetic, technical, and biotechnological advantages over native oleaginous bacteria, we aimed to construct a metabolically engineered *E. coli* strain capable of accumulating high levels of triacylglycerol (TAG) and evaluate its neutral lipid productivity during high cell density fed-batch fermentations.

**Results:**

The *Streptomyces coelicolor* TAG biosynthesis pathway*,* defined by the acyl-CoA:diacylglycerol acyltransferase (DGAT) Sco0958 and the phosphatidic acid phosphatase (PAP) Lppβ, was successfully reconstructed in an *E. coli* diacylglycerol kinase (*dgkA*) mutant strain. TAG production in this genetic background was optimized by increasing the levels of the TAG precursors, diacylglycerol and long-chain acyl-CoAs. For this we carried out a series of stepwise optimizations of the chassis by 1) fine-tuning the expression of the heterologous SCO0958 and *lpp*β genes, 2) overexpression of the *S. coelicolor* acetyl-CoA carboxylase complex, and 3) mutation of *fadE,* the gene encoding for the acyl-CoA dehydrogenase that catalyzes the first step of the β-oxidation cycle in *E. coli*. The best producing strain, MPS13/pET28-0958-ACC/pBAD-LPPβ rendered a cellular content of 4.85% cell dry weight (CDW) TAG in batch cultivation. Process optimization of fed-batch fermentation in a 1-L stirred-tank bioreactor resulted in cultures with an OD_600nm_ of 80 and a product titer of 722.1 mg TAG L^-1^ at the end of the process.

**Conclusions:**

This study represents the highest reported fed-batch productivity of TAG reached by a model non-oleaginous bacterium. The organism used as a platform was an *E. coli* BL21 derivative strain containing a deletion in the *dgkA* gene and containing the TAG biosynthesis genes from *S. coelicolor.* The genetic studies carried out with this strain indicate that diacylglycerol (DAG) availability appears to be one of the main limiting factors to achieve higher yields of the storage compound. Therefore, in order to develop a competitive process for neutral lipid production in *E. coli*, it is still necessary to better understand the native regulation of the carbon flow metabolism of this organism, and in particular, to improve the levels of DAG biosynthesis.

**Electronic supplementary material:**

The online version of this article (doi:10.1186/s13068-014-0172-0) contains supplementary material, which is available to authorized users.

## Background

**Figure 1:**
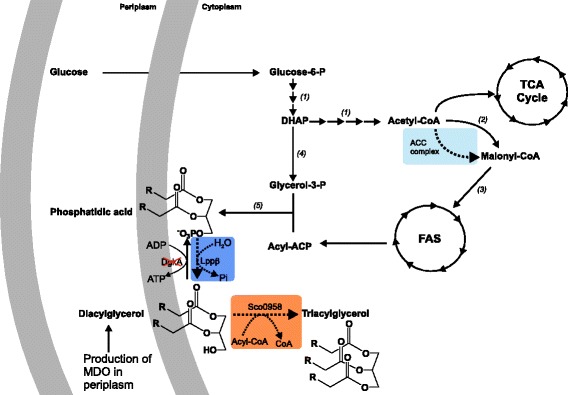
**Engineering** ***E. coli*** **for TAG biosynthesis.** Heterologous proteins expressed in *E. coli* are highlighted in color, and the knockout of the *E. coli* DgkA is labeled with a red cross. Sco0958, Lppβ, and ACC correspond to diacylglycerol acyltransferase, phosphatidic acid phosphatase, and acetyl-CoA carboxylase enzymes from *S. coelicolor*, respectively. *(1)* Glycolytic pathway; *(2)* acetyl-CoA carboxylase; *(3)* malonyl-CoA:ACP transacylase; *(4)* glycerol-3-P dehydrogenase; *(5)* glycerol-3-P and lysophosphatidic acid acyltransferases; DHAP, dihydroxyacetone phosphate; FAS, fatty acid synthase; TCA, tricarboxylic acid; MDO, membrane-derived oligosaccharide.

Triacylglycerols (TAGs) are the most common lipid-based energy reserve in animals, plants, and eukaryotic microorganisms [1]. These neutral lipids, traditionally sourced from plant oils, have widespread applications in industry and healthcare, in addition to their main use in food and feed purposes. In particular, about 20% of the plant oils produced today is utilized in certain applications of the oleochemical industry due to the lower downstream processing costs [2]. Furthermore, since the demand for oleochemicals is expected to grow, microbial production of free fatty acids (FAs) and FA-derived compounds such as TAGs, waxes, alkanes, or long-chain alcohols offers a great potential as an alternative to plant-derived products [1,3-5]. Compared to plant oils, microbial oils have many advantages; for example, they have a short life cycle, are less labor intensive, are not as affected by venue, season, and climate, and are easier to scale up [6]. The microbial production of oils also offers the possibility to reduce the competition with food feedstocks and the use of, for example, cellulose, lignin, hemicellulose, CO_2_, or other non-food carbon sources as raw material. Microorganisms that naturally synthesize TAG (like species of *Rhodococcus*, *Mycobacterium*, and *Streptomyces* genera) can reach high levels of this neutral lipid under specific growth conditions, as is the case of *R. opaccus* grown on gluconate medium which is capable of accumulating TAG accounting for up to 76% of the cell dry weight (CDW) [1,7]. Unfortunately, these microorganisms generally exhibit a rather slow growth rate and may require substantial and challenging genetic modifications for higher TAG productivity and/or for substrate utilization [8]. In this context, TAG production in *Escherichia coli* may become a valid alternative [9]. To date, a few recent studies have been published describing engineered pathways for this neutral lipid biosynthesis in *E. coli* [8,10,11]. These heterologous routes are based on the production and accumulation of the precursors, diacylglycerol (DAG) and fatty acyl-CoAs, which are esterified to synthesize TAG by the activity of a diacylglycerol acyltransferase (DGAT) (Figure 1). To raise the intracellular DAG concentration in *E. coli*, two alternatives have been described: 1) the overexpression of the native phosphatidylglycerol phosphate phosphatase PgpB [8], or the heterologous expression of the phosphatidate phosphatase Lppβ from *Streptomyces coelicolor* [12]; and 2) the generation of a knockout mutant in the *E. coli* diacylglycerol kinase gene, *dkgA.* Deletion of *dgkA* impairs the recycling of the DAG generated during the synthesis of membrane-derived oligosaccharides, thus leading to the accumulation of the DAG moiety [13,14].

So far, TAG production in *E. coli* could be considered a feasible development if the engineering process could lead to a microorganism that accumulates TAG efficiently, while maintaining its fast growth properties [9]. Thus, the aim of this work was to construct a recombinant *E. coli* strain that could synthesize and store high levels of this neutral lipid. To do this we selected the two dedicated TAG biosynthesis enzymes from *S. coelicolor*, the phosphatidic acid phospatase Lppβ and the acyl-CoA:diacylglycerol acyltransferase Sco0958, and followed a modular engineering strategy to optimize their expression levels in the heterologous host. Further genetic modifications introduced in the TAG producer strain allowed us to obtain an *E. coli* capable of accumulating the highest levels of TAG reported for fed-batch fermentations.

## Results and discussion

### Impact of the *S. coelicolor* PAP activity in TAG biosynthesis in *E. coli*

**Figure 2:**
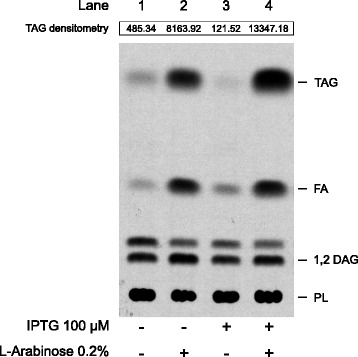
**Radio-thin layer chromatography (TLC) of** ***E. coli*** **MPS11/pBAD-0958/pET28-LPPβ** **total lipid extracts.** Cultures of *E. coli* were grown as described in Methods, and expression of Sco0958 and Lppβ enzymes was induced with L-arabinose and IPTG, respectively. Each lane contains the lipid extracts of 1 mg cell dry mass, and the values indicated above represent the densitometry measurement of TAG expressed in arbitrary units. TAG, triacylglycerol; FA, fatty acids; DAG, diacylglycerol; PL, phospholipids.

In previous work Comba *et al.* demonstrated that the heterologous co-expression of the *S. coelicolor* DGAT (acyl-CoA:diacylglycerol acyltransferase) Sco0958 and the PAP (phosphatidic acid phosphatase) Lppβ, in an *E. coli* BL21 (DE3) strain leads to the production of TAG, while the sole expression of Sco0958 is unable to induce accumulation of this neutral lipid in the same genetic background [12]. On the other hand, Arabolaza *et al.* showed that the expression of the DGAT Sco0958 in an *E. coli* strain containing a deletion in the *dgkA* gene is sufficient to induce TAG accumulation [15], suggesting that DAG is the limiting metabolite for TAG biosynthesis in *E. coli*. To test if DAG was still a limiting factor in a *dgkA* background, we evaluated the impact of Lppβ in TAG biosynthesis in a BL21 Δ*dgkA* strain - named strain MPS11 - in the presence of Sco0958. To do this we transformed the MPS11 strain with plasmids pBAD-0958, containing the *SCO0958* gene under the P_BAD_ promoter, and pET28-LPPβ, which contains the *lpp*β gene under the T7 promoter. As shown in Figure 2, the sole expression of *SCO0958* in the MPS11 strain was sufficient to promote accumulation of significant levels of TAG (Figure 2, lane 2). However, co-expression of both the Sco0958 and Lppβ enzymes increases TAG production by 1.6-fold (Figure 2, lane 4 versus lane 2). These results indicate that DAG availability is crucial for obtaining higher neutral lipid yields and that the PAP activity of Lppβ does help to circumvent this bottleneck in the TAG biosynthesis pathway reconstructed in *E. coli* BL21. Interestingly, low levels of TAG biosynthesis were also detected under conditions of no induction of either *SCO0958* or *lpp*β, or when only *lpp*β expression was induced (Figure 2, lanes 1 and 3, respectively); this low level of TAG production may occur by the leaky expression of *SCO0958* from P_BAD_ or, as suggested by Rotering and Raetz, by the existence of a hitherto unrecognized metabolism of minor neutral lipids biosynthesis in *E. coli* [16]*.*

In this regard, the native *E. coli* phosphatidylglycerol phosphate phosphatase, PgpB, has been used as a surrogate PAP for TAG production [8]. Specifically, in a recent work Janssen and Steinbüchel described an *E. coli* K12 strain capable of producing 0.3% of the cellular dry mass of TAG by overexpressing PgpB and the fatty acyl-CoA synthase FadD from *E. coli* plus the DGAT AtfA from *Acinetobacter baylyi* [11]. Although this pathway was not reconstituted in a *dgkA* background, making a direct comparison with our approach somehow difficult, it is appropriate to highlight that PgpB is a broad substrate spectrum enzyme, as demonstrated by its *in vitro* phosphatase activities towards phosphatidylglycerol, phosphatidic acid, lysophosphatidic acid, diacylglycerol pyrophosphate, and undecaprenyl pyrophosphate [17-19], while Lppβ is a dedicated PAP involved in generating DAG for TAG biosynthesis in *S. coelicolor* [12]*.*

### Fine-tuning TAG biosynthesis genes expression

**Figure 3:**
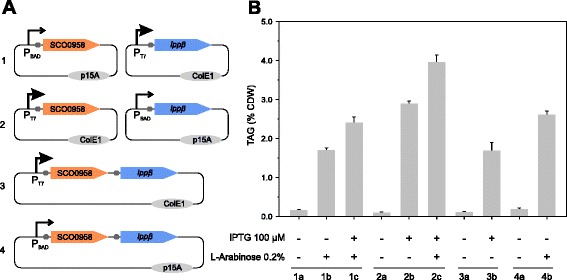
**Optimization of TAG biosynthesis in the MPS11 strain. A)** Schematic representation of the plasmid systems used for modulating SCO0958 and *lpp*β genes expression. The gray dot upstream of the target genes represents Shine-Dalgarno sequences and the size of the bended arrows, the relative strength of the inducible P_T7_ or P_BAD_ promoters. **B)** Cultures of *E. coli* MPS11 containing the different expression systems (1 to 4) were grown as described in Methods section, and expression of the respective genes was induced with L-arabinose and/or isopropyl-β-D-thiogalactopyranoside (IPTG). TAG content recovered from each of the cultures is expressed as percentage of cell dry weight mass (% CDW).

In order to optimize TAG production in our *E. coli* platform, we examined the use of alternative promoters, genes organization, and plasmid copy numbers to modulate the expression levels of *SCO0958* and *lpp*β and to evaluate their impact in TAG biosynthesis. To do this, we constructed several expression vectors derived from plasmids pET28a or pBAD33 harboring either *SCO0958* or *lpp*β as independent transcription units or as part of a bicistronic operon (Figure 3A). To analyze the levels of TAG produced by the individual expression systems, the corresponding plasmids were transformed into *E. coli* MPS11 and isolated clones of each of the four recombinant strains used for production studies in shake flasks. Figure 3B shows that the sole expression of *SCO0958* from the strong T7 promoter notably increases TAG content in the cell respective to its expression from the P_BAD_ promoter, demonstrating that under these conditions higher levels of the DGAT Sco0958 result in higher levels of TAG biosynthesis (Figure 3B, compare columns 1b and 2b). As expected, in all cases where the two genes of the system were induced, TAG accumulation was further improved, suggesting that the PAP Lppβ increases the levels of DAG, which is otherwise a limiting metabolite of the system. Maximal production of TAG was reached by co-expressing *SCO0958* from the strong T7 promoter and *lpp*β from P_BAD_ (Figure 3B, column 2c). A western blot analysis of Sco0958 and Lppβ expression for each recombinant strain constructed indicates that construction 2, condition c of Figure 3 represents the optimum protein levels to reach maximal TAG accumulation (Additional file 1: Figure S1). Unexpectedly, the coupled expression of the *SCO0958*-*lpp*β operon, either from P_T7_ or P_BAD_ promoters, was accompanied by a medium to low accumulation of TAG, suggesting that in either case the relationship between Sco0958 and Lppβ was not optimal (Figure 3B, columns 3b and 4b). In these single-plasmid systems, the levels of expression of both enzymes are lower compared to the situation where the *SCO0958* and *lpp*β genes are located in two different plasmids (Additional file 1: Figure S1), leading to a suboptimal activity of the pathway. Overall, these results support the notion that expression of *SCO0958* and *lpp*β has to be finely modulated in order to achieve optimal levels of TAG biosynthesis. Further studies will be necessary to better understand the basis of the transcription-translation dynamics of these genes in both expression systems.

**Figure 4:**
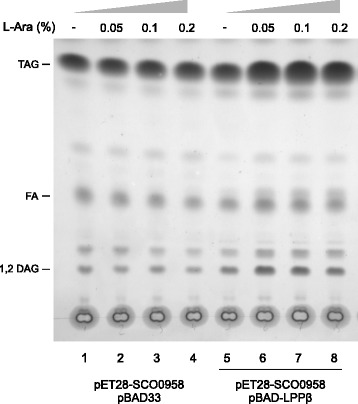
**TLC analysis of total lipid extracts from** ***E. coli*** **MPS11 derivative strains.** Cultures of *E. coli* were grown as described in Methods, and expression of Sco0958 and Lppβ enzymes was induced with IPTG and L-arabinose, respectively. pBAD33 was used as an empty plasmid control. Lipid pattern obtained after processing 1 mg cell dry mass visualized by Cu-phosphoric staining. L-Ara, L-arabinose; TAG, triacylglycerol; FA, fatty acids; DAG, diacylglycerol.

Curiously, although the P_BAD_ promoter is weaker than P_T7_ and the p15A origin of replication has a lower copy number than ColE1, the Lppβ protein levels in condition 2c are slightly higher than in condition 1c (Additional file 1: Figure S1). Moreover, when both proteins are expressed, the Sco0958 protein titer decreases compared to the situation where it is induced alone. This effect could be due to the high metabolic burden imposed on the cell when two, instead of one, proteins are expressed from strong promoters. In this sense, to further optimize the TAG-producing strain MPS11/pET28-0958/pBAD-LPPβ, we evaluated the inducer dose-response to find the best L-arabinose concentration to reach maximal TAG content. Cultures of the producer strain were grown in Luria-Bertani (LB) medium and induced with different concentrations of L-arabinose (Figure 4, lanes 5 to 8). TAG quantification carried out by LC-MS revealed that 0.1% of L-arabinose was the inducer concentration that leads to the highest TAG content. Furthermore, to evaluate a possible contribution from the catabolism of L-arabinose in TAG production, we included a control with an empty pBAD33 plasmid instead of the pBAD-LPPβ vector. In these cultures, increasing concentrations of L-arabinose had no effect on the final TAG content (Figure 4, lanes 1 to 4).

In summary, the systematic studies carried out towards optimizing the expression levels of the specific TAG biosynthesis genes led us to identify the most suitable strain, MPS11/pET28-0958/pBAD-LPPβ, and gene induction conditions, 100 μM IPTG and 0.1% L-arabinose, to reach a maximal TAG production of 4.15% CDW.

### Neutral lipid profile of engineered *E. coli* expressing Sco0958 or AtfA

**Figure 5:**
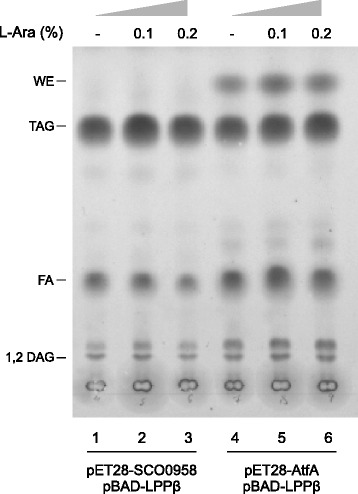
**Total lipid analysis of** ***E. coli*** **MPS11 strain expressing either the Sco0958 or the AtfA DGAT enzymes, in combination with Lppβ** **.** Cultures of *E. coli* were grown and analyzed as described in Methods. Lipid pattern obtained after processing 1 mg cell dry mass visualized by Cu-phosphoric staining. L-Ara, L-arabinose; WE, wax esters; TAG, triacylglycerol; FA, fatty acids; DAG, diacylglycerol.

The AtfA enzyme from *A. baylyi* was the first member of the thereafter designated WS/DGAT prokaryotic family of proteins [20]. Heterologous expression of this enzyme has been used for the production of fatty acid methyl esters, waxes, and TAG in *E. coli* [3,8,21]. In order to specifically evaluate the TAG biosynthesis capabilities of AtfA and compare it with our producer strain containing Sco0958 as a DGAT, we cloned the *atfA* gene in the pET28 expression vector, generating plasmid pET28-AtfA, and transformed it into an MPS11 derivative strain harboring the pBAD-LPPβ plasmid. As shown in Figure 5, AtfA expression led to a significant production of TAG, but also of an additional compound identified as wax esters by their retention factor on the TLC plates. This result agrees with previous reports where AtfA is confirmed to be capable of synthesizing butyl esters in *E. coli,* even in the absence of a supplemented alcohol [3]. Moreover, the levels of TAG achieved with AtfA were 10.6% lower than the ones obtained with Sco0958. Although expression of AtfA could be optimized to increase TAG titers, these results suggest that Sco0958 has a higher specificity than AtfA for TAG synthesis and, therefore, Sco0958 appears to be a better candidate for TAG production in *E. coli*.

### Metabolic engineering further improves TAG accumulation

To further improve TAG titers in our recombinant strain, we followed a classic metabolic engineering approach to enhance the pools of substrates of the TAG biosynthesis enzymes. To increase fatty acyl-CoA availability, we proceeded to engineer the fatty acid oxidation pathway of *E. coli* through the overexpression of *fadD* and the disruption of *fadE*. FadD activates free fatty acids into the metabolically active acyl-CoA thioesters [22], and FadE is an acyl-CoA dehydrogenase that catalyzes the first step of the β-oxidation cycle, the oxidation of an acyl-CoA to a 2-enoyl-CoA [23]. The co-expression of Sco0958 and Lppβ enzymes in a BL21 ∆*dgkA* ∆*fadE* strain - named the MPS13 strain - led to a 9% higher accumulation of TAG compared with its parental strain MPS11. Unexpectedly, overexpression of FadD in the ∆*dgkA* ∆*fadE* genetic background did not further increase the levels of TAG (Additional file 1: Table S1). This result suggests that under the conditions tested the native FadD activity is sufficient to activate the available FAs to their respective acyl-CoAs.

In order to further optimize TAG production in *E. coli,* we decided to overexpress proteins involved in key steps of the central carbon metabolism that could be working as bottlenecks in the carbon flow towards TAG biosynthesis. For this purpose we selected the MPS13/pBAD-LPPβ strain and transformed it with several pET28-based vectors for the co-expression of Sco0958 with the following selected targets: 1) GpsA, a glycerol 3-phosphate (G3P) dehydrogenase from *S. coelicolor,* which catalyzes the generation of G3P from the glycolysis intermediate dihydroxyacetone phosphate. This enzyme was selected based on previous results that showed that expression of a *Saccharomyces cerevisiae* G3P dehydrogenase in *Brassica napus* raised their seed oil content by 40% [24]; 2) a second copy of *SCO0958*; 3) FadR, the dual transcriptional regulator of fatty acid metabolism in *E. coli* [25,26]. FadR is a repressor of the β-oxidation genes and an activator of the fatty acid biosynthesis genes *fabA* and *fabB*. The selection of FadR was based on previous results where it was shown that its overexpression in *E. coli* led to a significant increase in fatty acid content [27]; 4) The acetyl-CoA carboxylase complex (ACC) from *S. coelicolor*, constituted by three subunits AccA2, AccB, and AccE, which generates the malonyl-CoA utilized for fatty acid chain elongation [28,29]. The analysis of TAG accumulation in each of the derivatives strains containing the combination of genes described above indicated that neither *gpsA*, nor the extra copies of *SCO0958* or *fadR* improved the levels of TAG obtained with the original system (4.52% CDW in MPS13/pET28-0958/pBAD-LPPβ, Additional file 1: Table S1). However, expression of the *S. coelicolor* ACC complex led to a 7.3% increase in TAG production, reaching a TAG content of 4.85% CDW (Additional file 1: Table S1). ACC complex overexpression is expected to increase FA synthesis, thus raising the levels of FA-derivative lipids. In this context, ACC enzymes could have a double role in TAG synthesis precursor supply, since it would increase the availability of DAG - through the increase of PA - and/or of the fatty acyl-CoA precursors.

Several metabolic engineering efforts have been carried out in *E. coli* in order to improve FA production, as they are potential precursors for FA-derived hydrocarbons [4,5,9,10,27,30-37]. However, little has been done in this host to channel these FAs towards TAG biosynthesis. In this sense, Lin *et al.* constructed an *E. coli* BL21 derivative strain carrying multiple combinations of deletions in genes involved in central carbon flux [10]. Although they successfully increased FA availability in some of their mutant strains, they did not obtain a concomitant increase in TAG content when the TAG biosynthesis enzymes were overexpressed [10]. These results confirm that additional bottlenecks must be overcome to achieve higher TAG titers. Interestingly, Lin *et al.* did not use a PAP enzyme to improve DAG availability in their hosts, which according to our results is the main limiting metabolite for TAG biosynthesis.

### Fed-batch cultivation for high yield TAG production

Based on the results obtained in our batch culture studies, we selected the MPS13/pET28-0958-ACC/pBAD-LPPβ strain for the optimization of TAG production in high cell density fermentations. Before initiating these studies, we introduced one additional mutation in the producing strain in order to prevent L-arabinose metabolization and thus avoid permanent feeding of the inducer; to do this we knocked out the complete *araBAD* operon in MPS13, giving rise to the BL21 ∆*dgkA* ∆*fadE* ∆*araBAD,* named the MPS15 strain. Fed-batch cultures were performed in minimal medium, using glucose as the carbon source, at 37°C. Heterologous protein production was induced by addition of 1 mM IPTG and L-arabinose 0.1% at OD_600nm_ about 60 after shifting the temperature of the process to 23°C to improve protein solubility. Glucose was kept limiting throughout the process in order to avoid overflow of the tricarboxylic acid (TCA) cycle and the concomitant production of acetate. Dissolved oxygen was maintained above 40% to prevent anaerobic fermentation of the carbon source. Samples were taken periodically for neutral lipids analysis.

**Figure 6:**
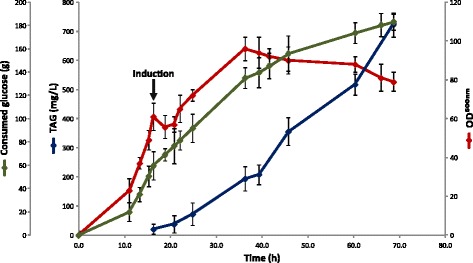
**Fed-batch cultivation of MPS15/pET28-0958-ACC/pBAD-LPPβ** **strain.** *E. coli* strain was cultured as described in Methods, and samples were taken periodically for OD, glucose, and neutral lipids analysis.

Figure 6 shows the progression of the fed-batch cultures carried out in this study. The complete process took 68 h, and at the end of the fermentation, the MPS15/pET28-0958-ACC/pBAD-LPPβ strain was able to synthesize 722.1 mg TAG L^-1^, reaching a cell TAG content of 2.83% CDW. Under these conditions, the volumetric productivity was 10.54 mg TAG L^-1^ h^-1^ at the end of the process. Furthermore, the FA composition of the resulting *E. coli* single cell oil was also analyzed and is described in Additional file 1: Table S2. The most abundant triglyceride species reflect the composition of total *E. coli* FA, as they are composed mainly of saturated and monounsaturated C16 and C18 FAs. Similar results were reported by Rucker *et al.* by analyzing the FA composition of *E. coli* TAG grown in batch cultures [8].

To our knowledge, the only other study where TAG production in *E. coli* was evaluated in fed-batch fermentors is that from Janssen and Steinbüchel [11]. In their work the authors used an *E. coli* K12 derivative strain that reached a final OD_600nm_ of 13.2 after 68 h and produced 532 mg L^-1^ TAG, with a maximal TAG content of 8.5% CDW under limited nitrogen conditions [11].

Although the maximal TAG content of strain MPS15/pET28-0958-ACC/pBAD-LPPβ obtained during the fermentation process was significantly lower than the ones obtained in batch assays, the cultures grown in the reactors were still able to produce the highest TAG titers reported for *E. coli* until now. It is difficult to predict the cause(s) of this striking difference in TAG accumulation between the two different growth systems, although we could speculate that the use of minimal medium in the reactors, instead of LB, could account for such a difference. However, we also observed that batch cultures of the selected strain carried out in minimal medium supplemented with 5 g L^-1^ glucose - which is below the catabolite repression threshold - have the same production levels as the batch cultures grown in LB medium (data not shown). Therefore, other bottlenecks or limitations appear to exist during fermentation that do not allow higher accumulation of the neutral lipid. We speculate that further improvements or optimization of the fermentation process should let us reach the production levels that were achieved in batch cultures.

## Conclusions

In this work we described the successful reconstruction of the *S. coelicolor* TAG biosynthesis pathway in the model heterologous host *E. coli.* By genetically engineering the *E. coli* producer strain, we improved the final TAG content up to 4.85% CDW in batch cultures. Additionally, the fed-batch microbial fermentation was also optimized, achieving a maximum yield of 722.1 mg TAG L^-1^ and a volumetric productivity of 10.54 mg TAG L^-1^ h^-1^. These results represent the highest reported fed-batch productivity of TAG reached by a non-oleaginous heterologous bacterium. However, the titer of TAG produced by this recombinant *E. coli* is still low compared with those of natural oleaginous producers such as *Rhodococcus* sp. Considering the productivities of TAG reported for fed-batch fermentations of *Rhodococcus opaccus*, much basic research is needed in the *E. coli* system in order to efficiently convert the high levels of FA that this organism can produce into higher concentrations of TAG or of other high-value hydrocarbons of industrial interest.

## Methods

### Media and growth conditions

*E. coli* strains were routinely grown either on solid or in liquid LB medium at 37°C and supplemented when needed with the following antibiotics: 100 μg mL^-1^ ampicillin (Ap), 50 μg mL^-1^ kanamycin (Km), or 20 μg mL^-1^ chloramphenicol (Cm). For auxotrophy tests, M9 mineral media supplemented with either 0.2% glucose, 0.2% L-arabinose, or 0.1% oleate/0.4% Brij 58 were used. For solid media, 1.5% agar was added before autoclaving.

The minimal medium for fed-batch fermentation consisted of 0.5 g L^-1^ yeast extract, 20.8 g L^-1^ KH_2_PO_4_, 3 g L^-1^ (NH_4_)_2_HPO_4_, and 4 g L^-1^ NaH_2_PO_4_. After autoclaving, it was supplemented with 50 μg mL^-1^ Km, 20 μg mL^-1^ Cm, 1.5 mL of trace element solution per liter, and the corresponding amount of carbon source solution. The trace element solution contained 50 mM FeCl_3_, 20 mM CaCl_2_, 10 mM ZnSO_4_, 10 mM MnCl_2_, 2 mM CoCl_2_, 2 mM CuCl_2_, 2 mM NiCl_2_, 2 mM H_3_BO_3_, 2 mM Na_2_MoO_4_, and 2 mM Na_2_SeO_3_. The carbon source solution used contained 600 g L^-1^ glucose and 15 g L^-1^ MgSO_4_.7H_2_O.

### Strain construction

All the strains used in this study are listed in Table 1. The *E. coli* MPS11 strain was constructed by P1 transduction of the *dgkA*::*Km* marker from the JW4002 strain [38] to BL21 and subsequent FLP recombinase-mediated excision of the kanamycin cassette as described by Datsenko and Wanner [39]. The MPS13 strain was constructed by P1 transduction of the *fadE*::*Km* marker from the JW5020 strain [38] to MPS11 and subsequent FLP recombinase-mediated excision of the kanamycin cassette [39]. The MPS14 strain was constructed by the one-step inactivation of *E. coli* chromosomal genes described by Datsenko and Wanner [39]. For this, araBAD_F/araBAD_R oligonucleotides were used to amplify the Km cassette of the pKD13 plasmid. The resulting DNA fragment was used to transform MPS13/pKD46 electrocompetent cells to replace *araBAD* genes. The kanamycin resistance cassette of strain MPS14 was deleted by FLP recombinase-mediated excision, yielding the MPS15 strain [39]. Deletions of *dgkA* and *fadE* genes were checked by PCR using dgkA_F/R and fadE_F/R primer pairs. Deletion of *araBAD* genes was checked by PCR using ara_check_F/ara_check_R and araA_wt_F/ara_check_R primer pairs. Knockout of *fadE* and *araBAD* genes was also confirmed by growing the strains in M9 plates with glucose, arabinose, or oleate as sole carbon sources.

**Table 1:** **Strains and plasmids**

**Strain or plasmid**	**Description**	**Reference**
**Strains**		
DH5α	*E. coli K12 F* ^*-*^ *lacU169 (*Φ*80lacZ*∆*M15) endA1 recA1 hsdR17 deoR supE44 thi-1 l2 gyrA96 relA1*	[40]
BL21 (DE3)	*E. coli F* ^*-*^ *ompT gal dcm lon hsdS*_*B*_ *(r*_*B*_^*-*^ *m*_*B*_^*-*^ *)* λ*(DE3)*	Novagen
MPS10	BL21 (DE3) ∆*dgkA*::*Km*; Km^R^	This study
MPS11	BL21 (DE3) ∆*dgkA*	This study
MPS12	BL21 (DE3) ∆*dgkA* ∆*fadE*::*Km*; Km^R^	This study
MPS13	BL21 (DE3) ∆*dgkA* ∆*fadE*	This study
MPS14	BL21 (DE3) ∆*dgkA* ∆*fadE* ∆*araBAD*::*Km*; Km^R^	This study
MPS15	BL21 (DE3) ∆*dgkA* ∆*fadE* ∆*araBAD*	This study
**Plasmids**		
pET28a	Vector for expression of N-terminal His-tagged proteins under the strong T7 promoter; Km^R^	Novagen
pBAD33	Vector for recombinant protein expression under the control of the *P*_*BAD*_ promoter; Cm^R^	[41]
pKD13	Template plasmid for amplification of the FRT-flanked kanamycin cassette; Ap^R^ Km^R^	[39]
pKD46	Temperature-sensitive replication plasmid for Red recombinase expression; Ap^R^	[39]
pCP20	Temperature-sensitive replication plasmid for thermal induction of FLP synthesis; Cm^R^ Ap^R^	[42]
pCR®-BluntII-TOPO	Vector used for cloning of blunt PCR products; Km^R^	Invitrogen
pBAD0958	pBAD33 carrying the *SCO0958* ^His^ gene under the control of *P*_*BAD*_ promoter; Cm^R^	[15]
pET28-LPPβ	pET28 carrying the *SCO1753* ^His^ gene under the control of T7 promoter; Km^R^	[12]
pBAD-LPPβ	pBAD33 carrying the *SCO1753* ^His^ gene under the control of *P*_*BAD*_ promoter; Cm^R^	[12]
pTR257	pET28 carrying the *SCO0958* ^His^ gene under the control of T7 promoter; Km^R^	[15]
pET28-0958- LPPβ	pET28 carrying the *SCO0958* ^His^-*SCO1753* ^His^ operon under the control of T7 promoter; Km^R^	This study
pBAD-0958- LPPβ	pBAD33 carrying the *SCO0958* ^His^-*SCO1753* ^His^ operon under the control of *P*_*BAD*_ promoter; Cm^R^	This study
pET28-AtfA	pET28 carrying the *atfA* ^His^ gene under the control of T7 promoter; Km^R^	This study
pET28-FadD	pET28 carrying the *fadD* ^His^ gene under the control of T7 promoter; Km^R^	This study
pET28-0958- FadD	pET28 carrying the *SCO0958* ^His^-*fadD* ^His^ operon under the control of T7 promoter; Km^R^	This study
pCC01	pET28 carrying the *accA2* ^His^ gene under the control of T7 promoter; Km^R^	[43]
pET28-AccA2-AccE	pET28 carrying the *accA2* ^His^-*accE* ^His^ operon under the control of T7 promoter; Km^R^	This study
pET28-AccA2-AccE-AccB	pET28 carrying the *accA2* ^His^-*accE* ^His^-*accB* ^His^ operon under the control of T7 promoter; Km^R^	This study
pET28-0958-ACC	pET28 carrying the *SCO0958* ^His^-*accA2* ^His^-*accE* ^His^-*accB* ^His^ operon under the control of T7 promoter; Km^R^	This study
pET28-GpsA	pET28 carrying the *gpsA* ^His^ gene under the control of T7 promoter; Km^R^	This study
pET28-0958-GpsA	pET28 carrying the *SCO0958* ^His^-*gpsA* ^His^ operon under the control of T7 promoter; Km^R^	This study
pET28-FadR	pET28 carrying the *fadR* ^*His*^ gene under the control of T7 promoter; Km^R^	This study
pET28-0958-FadR	pET28 carrying the *SCO0958* ^His^-*fadR* ^*His*^ operon under the control of T7 promoter; Km^R^	This study
pET28-0958-0958	pET28 carrying the *SCO0958* ^His^-*SCO0958* ^His^ operon under the control of T7 promoter; Km^R^	This study

### Plasmid construction

All the plasmids used in this study are listed in Table 1. The oligonucleotides used for PCR are listed in Table 2. The pET28-0958-LPPβ vector was constructed by ligation of the XbaI/HindIII digestion fragment of pET28-LPPβ into SpeI/HindIII restriction sites of pTR257. The XbaI/HindIII digestion fragment was then cloned in XbaI/HindIII sites of pBAD33, yielding plasmid pBAD0958-LPPβ.

**Table 2:** **PCR oligonucleotides**

**Name**	**Sequence (5′-3′)**	**Reference**
dgkA_F	AGGATCTGCCGGAATAGACTTGCTT	This study
dgkA_R	AGCGCCTTCAGATGTTCTTCAGCC	This study
fadE_F	CAAAAGCGAGAAGTACGGGCAGGTG	This study
fadE_R	GCTTTCGATTGATGGTAAAACGGTG	This study
araBAD_F	CTCGATTTTGGCAGTGATTCTGTGCGAGCTTTGGCGGTGG**CTGTCAAACATGAGAATTAA**	This study
araBAD _R	CCGTAATATGCCTTCGCGCCATGCTTACGCAGATAGTGTT**GTGTAGGCTGGAGCTGCTTC**	This study
ara_check_F	TAAACGAGTATCCCGGCAGCAGGGG	This study
ara_check _R	CGGGAATAAACGCCACGGACTCTTG	This study
araA_wt_F	AAGTGTATTACGGGTTTCGTCGCTA	This study
atfA_F	*CATATG*CGCCCATTACATCCGATTG	This study
atfA_R	*ACTAGT*CCTTTAGTTTTATCTGATA	This study
fadD_F	*GCTAGC*AAGAAGGTTTGGCTTAACC	This study
fadD_R	*ACTAGT*CTCAGGCTTTATTGTCCAC	This study
gpsA_F	*CATATG*AGCAAGCCGGTCAAGGCGG	This study
gpsA_R	*ACTAGT*GCGGGTTGCGCGGGGGGTC	This study
fadR_F	*CATATG*GTCATTAAGGCGCAAAGCC	This study
fadR_R	*ACTAGT*ATGGGAAATCTGTAAAAAC	This study
accB_F	*CATATG*ACCGTTTTGGATGAGGCGC	This study
accB_R	*ACTAGT*TCACTGCGGCGGGTTGCCG	This study
accE_F	*CATATG*TCCCCTGCCGACATCCGCG	This study
accE_R	*ACTAGT*TCAGCGCCAGCTGTGCGGG	This study

Restriction sites are shown in italics. Homologous sequences to pKD13 plasmid are shown in bold.

The *atfA* gene [GenBank:2879218] was amplified by PCR from *Acinetobacter sp*. ADP1 genomic DNA using the atfA_F/atfA_R primer pair. The *fadD* [GenBank:8182496] and *fadR* [GenBank:8181871] genes were amplified by PCR from *E. coli* BL21 genomic DNA using the fadD_F/fadD_R and fadR_F/fadR_R primer pairs, respectively. The *gpsA* [GenBank:1101000], *accB* [GenBank:1100975], and *accE* [GenBank:1100976] genes were amplified by PCR from *S. coelicolor* genomic DNA using the gpsA_F/gpsA_R, accB_F/accB_R and accE_F/accE_R primer pairs, respectively. The resulting PCR products were cloned in the pCR®-BluntII-TOPO vector and checked by DNA sequencing (University of Maine DNA Sequencing Facility, Orono, ME, USA). The AtfA, FadR, GpsA, AccB, and AccE coding sequences were cloned in pET28a as NdeI/EcoRI digestion fragments, yielding plasmids pET28-AtfA, pET28-FadR, pET28-GpsA, pET28-AccB, and pET28-AccE, respectively. *fadD* was cloned as an NheI/EcoRI digestion fragment into pET28a, yielding plasmid pET28-FadD.

The pET28-AccA2-AccE plasmid was constructed by ligation of the XbaI/HindIII digest of pET28-AccE into SpeI/HindIII sites of pCC01. The XbaI/HindIII digestion fragment of pET28-AccB was then cloned into SpeI/HindIII sites of pET28-AccA2-AccE, yielding plasmid pET28-AccA2-AccE-AccB. This plasmid carries the coding sequences for the complete acetyl-CoA carboxylase complex of *S. coelicolor*.

pET28a derivative vectors carrying the FadD, AccA2-AccE-AccB, FadR, GpsA, and SCO0958 coding sequences were digested with XbaI/HindIII restriction enzymes, and the resulting fragments were cloned into SpeI/HindIII sites of pTR257, yielding plasmids pET28-0958-FadD, pET28-0958-ACC, pET28-0958-FadR, pET28-0958-GpsA, and pET28-0958-0958, respectively.

### Total lipid analysis

Batch cultures of *E. coli* strains were grown in LB media at 37°C until OD_600nm_ was about 0.6. Then, protein expression was induced by addition of IPTG and/or L-arabinose. When the *E. coli* strain harbored pET28-LPPβ and pBAD-0958 plasmids, L-arabinose was first added at OD_600nm_ 0.6 and IPTG was added 1 h later. After induction, the cultures were kept overnight at 23°C with agitation. For ^14^C labeled experiments 3 μCi [^14^C]-acetate (58.9 Ci/mol, Perkin Elmer) was added to 5 mL of culture, at the same time as the protein induction. Cultures were then normalized by CDW and total lipids were extracted as described by Bligh and Dyer [44].

The lipid extracts were dried and analyzed by TLC on silica gel 60 F254 plates (0 ± 2 mm, Merck), using the solvent systems hexane/diethylether/acetic acid (75:25:1, v/v/v) [16]. Lipid fractions were visualized by Cu-phosphoric staining and identified by comparing to the mobility of known standards. For ^14^C labeled lipids the radioactivity incorporated into each lipid fraction was detected by autoradiography using Carestream® Kodak® BioMax® MR films. All the TLC plates were digitalized and the spots were quantified using ImageJ v1.48 software.

### SDS-PAGE and immunoblot

SDS-PAGE and immunoblot analyses using nitrocellulose membranes were carried out using standard protocols [45,46]. For detection of the His-tagged proteins, mouse monoclonal anti-His antibodies (QIAGEN™) were used at a dilution of 1:1000. Anti-mouse IgG-alkaline phosphatase conjugates were used as secondary antibodies at a dilution of 1:3000. His-tagged proteins were visualized by immunoblots using chromogenic detection as described by the manufacturer.

### Triacylglycerol quantification by high resolution LC-MS

For quantification of triacylglycerides, the biomass was lyophilized and weighed, and 1 mg was processed for total lipid extraction as described by Bligh and Dyer [44]. 10 μg of cetyl palmitate was added before extraction as an internal standard for load control. Then 500 μL of chloroform were added to solubilize lipids and 50 μL of the lipid extract were diluted in an equal part of methanol. 5 μL of this solution were injected and separated on a ZORBAX Eclipse XDB-C8 column (3.0 × 50 mm, particle size =1.8 μm; Agilent, USA) using methanol supplemented with 5 mM ammonium acetate as the mobile phase. The outlet of the liquid chromatograph was connected to a micrOTOF mass spectrometer (Bruker Daltonik, Bremen, Germany) operating in the positive-ion mode, and the data was acquired online in the mass range m/z 300-1500. TAGs were detected as ammonium adducts in the range of 6-20 min of the chromatography run. A calibration curve was done using pure tripalmitin as a standard. The TAG concentration in the samples was calculated by the linear regression equation obtained from the calibration curve.

### Fed-batch cultivation

A BioFLO 110 2 Lt Fermentor/Bioreactor (New Brunswik Scientific®) containing 0.9 L of minimal medium described above was autoclaved at 121°C for 30 min. After sterilization, 50 mg Km, 20 mg Cm, 20 g glucose, 0.5 g MgSO_4_.7H_2_O, and 1.5 mL trace element solution were added aseptically to the bioreactor. The reactor temperature, pH, and dissolved oxygen (DO_2_) were monitored using specific probes (Mettler-Toledo®). The temperature was maintained at 37°C or 23°C using a heat blanket and cooling water. The reactor pH was maintained at 7.0 ± 0.1 by the addition of 15% (v/v) NH_4_OH. When the ammonium concentration reached 200 mM, 15% NH_4_OH was replaced by 5 M NaOH. Agitation was provided by a double six-bladed impeller with the stir speed set at 700 rpm, and the gas flow was alternated between air and oxygen to ensure that the DO_2_ content did not decrease below 40% saturation. The gas inflow rate was maintained at 0.33 vvm. Foaming was controlled by the addition of Antifoam A (Code A5633, Sigma-Aldrich®). The glucose and ammonium concentrations were measured with enzymatic kits from Wiener Lab®, codes 1400101 and 1810050, respectively. Inoculation was carried out at 37°C with 100 mL of seed culture at OD_600nm_ approximately 2. When glucose in the initial medium was depleted at 12 h after inoculation (OD_600nm_ about 20), a nutrient feed consisting of the described carbon source solution was initiated at a fixed rate of 0.2 mL/min. When the culture reached OD_600nm_ about 60, 16 h after inoculation, the temperature was reduced to 23°C, the nutrient feed rate was shifted to a fixed rate of 0.1 mL/min, and heterologous protein expression was induced by the addition of 100 μM IPTG and 0.1% (w/v) L-arabinose. The culture was allowed to continue until maximum TAG production was reached (68.5 h). TAG production (mg TAG/L), at a given time point, was calculated from the TAG content of the corresponding sample, considering the current OD_600nm_ and volume of the sample, and the current volume of the fed-batch culture at this time point.

## Additional file

Additional file 1: Figure S1. Western blot analysis of Sco0958 and Lppβ in the strains described in Figure 3. Protein extracts were resolved by SDS-PAGE in a 12% polyacrylamide gel and detected by immunoblotting using anti-His antibodies as described in the Methods section. Lane 1, MPS11; lanes 2 and 3, MPS11/pBAD-0958/pET28-LPPβ; lanes 4 and 5, MPS11/pET28-0958/pBAD-LPPβ; lane 6, MPS11/pET28-0958-LPPβ; lane 7, MPS11/pBAD-0958-Lppβ. Table S1. Cell TAG content of different *E. coli* strains determined by high resolution LC-MS. Table S2. *E. coli* triglyceride fatty acid relative composition.

## Abbreviations

Ara: arabinose
CDW: cell dry weight
DAG: diacylglycerol
DGAT: diacylglycerol acyltransferase
FA: fatty acid
IPTG: isopropyl-β-D-thiogalactopyranoside
LC-MS: liquid chromatography coupled to mass spectrometry
OD: optical density
PAP: phosphatidic acid phosphatase
PL: phospholipid
TAG: triacylglycerol, triglyceride
TLC: thin layer chromatography
