# Supplementary material for: Engineering a Streptomyces coelicolor biosynthesis pathway into Escherichia coli for high yield triglyceride production
Source: Biotechnol Biofuels. 2014 Dec 24;7:172. doi: 10.1186/s13068-014-0172-0 (PMC4295399; doi:10.1186/s13068-014-0172-0)
Supplement: Additional file 1: Figure S1. — Western blot analysis of Sco0958 and Lppβ in the strains described in Figure 3. Protein extracts were resolved by SDS-PAGE in a 12% polyacrylamide gel and detected by immunoblotting using anti-His antibodies as described in the Methods section. Lane 1, MPS11; lanes 2 and 3, MPS11/pBAD-0958/pET28-LPPβ; lanes 4 and 5, MPS11/pET28-0958/pBAD-LPPβ; lane 6, MPS11/pET28-0958-LPPβ; lane 7, MPS11/pBAD-0958-Lppβ. Table S1. Cell TAG content of different E. coli strains determined by high resolution LC-MS. Table S2. E. coli triglyceride fatty acid relative composition. [file 13068_2014_172_MOESM1_ESM.docx]

**ADDITIONAL FILE**

**Engineering a *Streptomyces coelicolor* biosynthesis pathway into *Escherichia coli* for high yield triglyceride production**

**Santiago Comba, Martín Sabatini, Simón Menendez-Bravo, Ana Arabolaza and Hugo Gramajo**

**Figure S1. Western blot analysis of Sco0958 and Lppβ in the strains described in Figure 3.** Protein extracts were resolved by SDS-PAGE in a 12% polyacrylamide gel and detected by immunoblotting using anti-His antibodies as described in the Methods section. Lane 1, MPS11; Lanes 2 and 3, MPS11/pBAD-0958/pET28-LPPβ; Lanes 4 and 5, MPS11/pET28-0958/pBAD-LPPβ; Lane 6, MPS11/pET28-0958-LPPβ; Lane 7, MPS11/pBAD-0958-Lppβ.

**
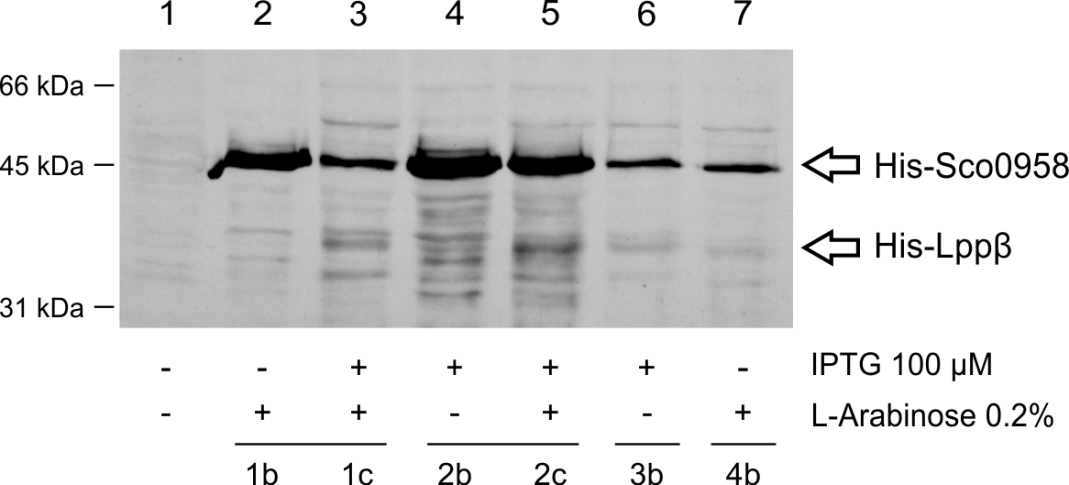
**

Table S1. Cell TAG content of different *E. coli* strains determined by High Resolution LC-MS

|  | Relevant genetic background | P_BAD_ expressed gene | PT7 expressed gene(s) | TAG (%CDW) |
| --- | --- | --- | --- | --- |
| 1 | Δ*dgkA* | *lppβ* | SCO0958 | 4.15 ± 0.05 |
| 2 | Δ*dgkA* Δ*fadE* | *lppβ* | SCO0958 | 4.52 ± 0.08 |
| 3 | Δ*dgkA* Δ*fadE* | *lppβ* | SCO0958-*fadD* | 3.9 ± 0.1 |
| 4 | Δ*dgkA* Δ*fadE* | *lppβ* | SCO0958-*gpsA* | 4.1 ± 0.2 |
| 5 | Δ*dgkA* Δ*fadE* | *lppβ* | SCO0958-SCO0958 | 4.45 ± 0.07 |
| 6 | Δ*dgkA* Δ*fadE* | *lppβ* | SCO0958-*fadR* | 2.9 ± 0.3 |
| 7 | Δ*dgkA* Δ*fadE* | *lppβ* | SCO0958-*accA2-accE-accB* | 4.85 ± 0.08 |

**Table S2. *E. coli* triglyceride fatty acid relative composition.**

| Triglyceride FA composition | Relative TAG content^a^ |
| --- | --- |
| 16:0/16:1/18:1 | 23.4 ± 0.9% |
| 16:0/18:1/18:1 | 15.0 ± 0.8% |
| 16:1/18:1/18:1 | 11.3 ± 0.6% |
| 16:0/16:0/16:1 | 11.1 ± 0.2% |
| 18:1/18:1/18:1 | 8.2 ± 0.4% |
| 16:0/16:1/16:1 | 8.0 ± 0.9% |
| 16:0/16:0/18:1 | 7.3 ± 0.5% |
| 16:1/16:1/18:1 | 4.4 ± 0.5% |
| 16:0/16:1/14:0 | 3.5 ± 0.5% |
| 18:1/18:1/18:0 | 2.1 ± 0.2% |
| Others^b^ | 5,7 ± 0.9% |

^a^ Values represent the mean and S.D. of triplicate determinations.

^b^ Includes minor TAG species: 16:0/16:0/16:0, 16:0/18:1/18:0, 16:1/16:1/14:0, 16:0/16:0/14:0, 16:0/16:1/12:0, 16:1/16:1/16:1, 16:0/16:0/12:0, 16:1/16:1/12:0, 16:1/14:0/12:0,14:0/14:0/14:0.
